# Characterization of occupational accidents occurred at the Occupational Health Referral Center of the state of Amapá between 2007 and 2016

**DOI:** 10.47626/1679-4435-2020-526

**Published:** 2021-04-30

**Authors:** Gilberto Tavares dos Santos Junior, Maria Helena Mendonça Araújo, Giorgio da Silva Araújo, Bráulio Erison França dos Santos, Raimundo Rodrigues da Costa Junior

**Affiliations:** 1 Faculdade de Medicina, Universidade Federal do Amapá, Macapá, AP, Brazil

**Keywords:** occupational accidents, biological material, occupational health

## Abstract

**Introduction:**

Occupational accidents involving biological material have consequences that range from physical damage to public expenses. The study of this topic may help evaluate conducts and form preventive measures.

**Objectives:**

To characterize the notifications of occupational accidents involving biological material that occurred between 2007 and 2016 in the state of Amapá, Brazil.

**Methods:**

This is an observational, retrospective, and descriptive study with a data analysis that quantified occupational accidents with biological material reported between January 2007 and December 2016 and analyzed the most prevalent risk factors.

**Results:**

Data were obtained from the Occupational Health Reference Center of the state of Amapá: 938 cases of occupational accidents with biological material were reported in the studied period. The main type of exposure was percutaneous (75.8%), the most common organic material was blood (68.4%), and the main causative agent was the hollow-bore needle (58.6%). Considering the reported cases, 80.8% of 745 individuals were vaccinated against hepatitis B and 2.4% of 252 individuals had positive anti-HIV. Regarding the clinical progression of the injured workers, in 91.9% of the cases these data were unknown or not recorded, and 47.4% of the patients who provided this information were discharged with no serological conversion.

**Conclusions:**

The main causes of occupational accidents were related to the use of sharps for intravenous drug administration and the inadequate disposal of this material, highlighting the need for stronger attention when performing these procedures. The high incidence of unknown/blank data hampered the correct serological follow-up of the patients and the epidemiological characterization of the accidents.

## INTRODUCTION

All professionals dealing with patients, biological waste management, and sharps are subject to exposure to biological material. The nursing team is more exposed to this problem due to their work activities.^1^ The occupational health policy of the Unified Health System (SUS) was strengthened with the creation of the National Network of Integral Attention to Occupational Health and the organization of Occupational Health Referral Centers (Centros de Referência em Saúde do Trabalhador [CEREST]).^2,3^ Occupational diseases listed as notifiable by the Ministry of Health^4^ should be registered in the Notifiable Diseases Information System (Sistema de Informação de Agravos de Notificação [SINAN]), which contains data on the person who suffered the accident and the circumstances involved. The care of a patient who has been a victim of a neddlestick or sharp injury should be provided in a welcoming environment, ensuring his or her privacy and providing information regarding the following steps. Chemoprophylaxis in case of infection by the human immunodeficiency virus (HIV) is recommended, depending on variables such as type of exposure, material, time since the accident, and serology of the exposed individual, with a preferential regimen of 3 drugs for 28 days.^5^ Prophylaxis for the hepatitis B virus (HBV) should be performed with vaccination and/or immunoglobulin, depending on the HBV serological status of the exposed and source individuals. No specific measures are established for exposure to the hepatitis C virus (HCV), and follow-up should be performed by the detection of the anti-HCV antibody for identifying serological conversion and treating cases of acute hepatitis C.^6^

Surveillance and prevention measures against accidents involving biological material are ensured by Regulatory Standard No. 32, which establishes guidelines for the implementation of a sharps injury prevention plan focused on the protection, safety, and health of health care workers. In addition, programs of continued education and personal protective equipment (PPE) use are essential in the prevention of these accidents.^7^ In this regard, we highlight the execution of this study of epidemiological characterization of the accidents occurred in the state of Amapá, Brazil, as a way of supporting the development of strategies that should be part of preventive policies, both at the municipal and state levels; this was performed by obtaining the sociodemographic profile and circumstances that influenced the occurrence of accidents involving biological material, as well as the professions and organic materials most involved in these accidents.

## METHODS

This is an observational, retrospective, and descriptive study, with a quantitative approach to the analysis of data from the CEREST of the state of Amapá; this center aims to promote the surveillance, rehabilitation and prevention of occupational accidents and participate in processes involving the notification and formation of the epidemiological profile of the main threats to worker health. The study was performed between January 2007 and December 2016 under the coverage of CEREST/AP and included, as target population, health professionals who suffered occupational accidents involving biological material in the state of Amapá, which were reported by SINAN and registered with code 20.9 in the International Statistical Classification of Diseases and Related Health Problems (ICD). The study considered all notifications of occupational accidents involving biological material that were reported during this period in health care institutions of the state of Amapá. The notification records of occupational accidents involving biological material have information on sociodemographic data of the injured worker (age, sex, residency, occupation, schooling), of the accident (use of PPE, causative agent, type of exposure, circumstances of the accident, organic material involved), and on the procedures that followed (serology of the source patient and injured worker regarding hepatitis B and C and HIV, immunization against hepatitis B, chemoprophylaxis with antiretroviral drugs).

In this population study, we analyzed data on 938 victims of occupational accidents involving biological material between 2007 and 2016. We applied descriptive statistics methods as our data reached the whole population and no inferences were required; the real parameters of the studied population were thus available in our study. Quantitative variables were presented through measures of central tendency and variation. Qualitative variables were presented as distributions of absolute and relative frequencies. Statistical processing was performed using BioEstat version 5.3.^8^ The observation of many diseases and accidents related to work activities allowed the definition of work as a health determinant. Therefore, health policies were developed with the aim of reducing these problems. In Brazil, the comprehensive attention to worker health refers to a segment of public health whose aim is to integrate actions involving the prevention of worker health problems and the promotion, assistance, and surveillance of occupational health.^9^

## RESULTS

Between 2007 and 2016, in the state of Amapá, 938 cases of occupational accidents involving biological material were reported. Among this series of cases, 745 (79.4%) were reported by the state capital Macapá and 193 (20.6%) were reported by other municipalities. The relative participation of Macapá was greater in 2019 (93%) and smaller in 2007 (40%) (Table 1).

**Table 1:** Notifications of occupational accidents involving biological material (n = 938) between 2007 and 2016 in the state of Amapá, Brazil, and relative participation of the state capital

	Amapá		Macapá		Other cities		Participation of Macapá
	n	%	n	%	n	%	
2007	20	2.1	8	40.0	12	60.0	0.9
2008	45	4.8	40	88.9	5	11.1	4.3
2009	71	7.6	66	93.0	5	7.0	7.0
2010	66	7.0	61	92.4	5	7.6	6.5
2011	71	7.6	52	73.2	19	26.8	5.5
2012	92	9.8	62	67.4	30	32.6	6.6
2013	147	15.7	102	69.4	45	30.6	10.9
2014	128	13.6	100	78.1	28	21.9	10.7
2015	123	13.1	105	85.4	18	14.6	11.2
2016	175	18.7	149	85.1	26	14.9	15.9
Total	938	100.0	745	79.4	193	20.6	

Regarding occupations, we observed that the nursing team corresponded to 54.8% (514) of the accidents, of which 469 were nursing technicians and 45 were nurses. Due to the more direct contact with patients and the administration of intravenous drugs, these professionals are more exposed to biological material.^1^ In this study, general workers represented 11% (103) of the accidents; students represented 6.1% (57); laboratory technicians represented 6% (56); oral health technicians accounted for 3.1% (29); administrative personnel accounted for 1.8% (17); and 3% (28) of the accidents did not have occupation data (unknown and/or blank). Physicians were divided into Type I (cardiologists, intensive care physicians, internists, and pediatricians) and Type II (general surgeons, anesthesiologists, vascular medicine physicians, plastic surgeons, gynecologists, and orthopedists). Each group corresponded to 2.2% (21) and 2.8% (26) of the total number of accidents involving biological material, respectively. Table 2 shows the relationships between the most injured professionals, age, and time of service at the moment of the occupational accident with biological material.

**Table 2:** Distribution of professions and time of service according to the occurrence of occupational accidents involving biological material (n = 938) between 2007 and 2016 in the state of Amapá, Brazil

Profession/occupation	Cases	%	Age (mean)	Time of service (years)
Nurse	45	4.8	36.4	3.0
Student	57	6.1	27.8	0,5
Nursing technician	469	50.0	36.4	2.5
Laboratory technician	56	6.0	31.9	3.0
Oral health technician	29	3.1	34.7	2.0
Administrative personnel	17	1.8	35.4	1.5
Type I physician	21	2.2	34.6	2.5
Type II physician	26	2.8	41.6	3.0
General worker	103	11.0	34.4	2.0
Others	87	9.3	32.6	2.0
Unknown	28	3.0	33.3	2.5

Four main axes are related to these exposure characteristics: type of exposure; organic material; causative agent of the accident; and use of PPE. The following characteristics were the most common in each of the axes: percutaneous exposure (75.8%); blood (68.4%) as the most common organic material; the hollow-bore needle (58.6%) as the main causative agent; and gloves (69.5%) as the most used piece of PPE. Table 3 shows the exposure characteristics. Even though workers were wearing gloves as PPE in 69.5% of accidents involving biological material, the use of masks (37.8%), aprons (24.2%), boots (16.3%), goggles (9.9%), and face shields (6.1%) was still scarce.

**Table 3:** Distribution of type of exposure and organic material according to the occurrence of occupational accidents involving biological material (n = 938) between 2007 and 2016 in the state of Amapá, Brazil

Exposure characteristics	n	%
Type of exposure		
Percutaneous	711	75.8
Mucosal	44	4.7
Uninjured skin	253	27.0
Injured skin	46	4.9
Other	13	1.4
Organic material		
Fluids with blood	24	2.6
Unknown/blank	173	18.4
Amniotic fluid	3	0.3
Ascitic fluid	1	0.1
Pleural fluid	2	0.2
Cerebrospinal fluid	2	0.2
Blood	642	68.4
Serum/plasma	3	0.3
Others	88	9.4
Agent		
Hollow-bore needle	550	58.6
Solid needle	109	11.6
Unknown/blank	78	8.3
Intravenous cannula	5	0.5
Blade/lancet	74	7.9
Glass	12	1.3
Others	110	11.7
Use of personal protective equipment		
Gloves	652	69.5
Apron	227	24.2
Goggles	93	9.9
Mask	355	37.8
Face shield	57	6.1
Boots	153	16.3

The main circumstances of accidents involving the exposure to biological material were the following: inadequate disposal (20.40%); intravenous drug administration (14.30%); venipuncture (8.4%); surgical procedure (7.3%); and intramuscular drug administration (5.8%). Other distributions are presented in Table 1. Considering the inadequate disposal of sharps, the most present causative agent was the hollow-bore needle (65.4%), and the main organic material was blood (59.2%).

**Figure 1:**
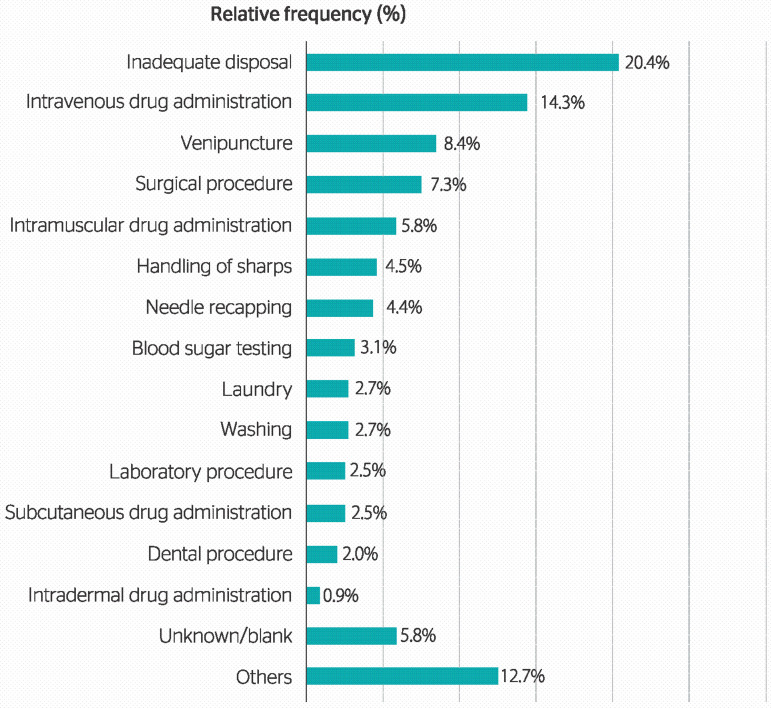
Distribution of circumstances of the accident according to the occurrence of occupational accidents involving biological material (n = 938) between 2007 and 2016 in the state of Amapá, Brazil.

The serological profiles of people who suffered occupational accidents involving biological material in the analyzed series were: vaccinated against hepatitis B (80.8% of 745 cases); positive anti-HIV (2.4% of 252 cases); positive HBsAg (1% of 122 cases); positive anti-HBs (11.2% of 107 cases); and positive anti-HCV (1% of 100 cases). We identified a high number of cases registered as unknown/blank for anti-HIV, HBsAg, anti-HBs, and anti-HCV: 686 (73.1%), 816 (87%), 831 (88.6%), and 838 (89.3%), respectively. As for the serological profile of the source patient, the most commonly identified characteristics were known source (63.3% of 780 answers); positive HBsAg (1.6% of 79 cases); positive anti-HIV (9.6% of 239 cases); positive anti-HBc (5.3% of 75 cases); and positive anti-HCV (7.4% of 81 cases). Regarding the recommendation for human immunoglobulin against hepatitis B and vaccination against this disease, 23 (2.5%) and 69 (7%) cases were reported.^10^ Considering therapies, only 188 cases (20%) received a chemoprophylaxis recommendation and 2% did not accept treatment; this was in accordance to other cases described by the literature.^11,12^ Cordeiro et al.^11^ and Corrêa et al.^12^ verified a refusal of 0.7% and 1.6% in their samples, respectively. Of these, 1 case presented positive anti-HIV, 9 individuals were injured when treating patients with positive anti-HIV status, and 30 answered this question as unknown/blank. Among the 16 individuals who accepted prophylaxis in this study, there were no records of positive anti-HIV for the injured professionals or source patients. However, 12 of them reported their source patients as unknown/blank (Table 4).

**Table 4:** Serological profile of source patients in occupational accidents involving biological material (n = 938) between 2007 and 2016 in the state of Amapá, Brazil

Source patient	n	%
Known source patient (n = 780)		
Yes	494	63.3
No	286	36.7
Ignored (n = 938)	158	16.8
HBsAg (source patient) (n = 79)		
Negative	75	94.9
Positive	4	5.1
Ignored (n = 938)	859	91.6
Anti-HIV (source patient) (n = 239)		
Negative	216	90.4
Positive	23	9.6
Ignored (n = 938)	699	74.5
Anti-HBc (source patient) (n = 75)		
Negative	71	94.7
Positive	4	5.3
Ignored (n = 938)	863	92.0
Anti-HCV (source patient) (n = 81)		
Negative	75	92.6
Positive	6	7.4
Ignored (n = 938)	857	91.4
Chemoprophylaxis recommendation (n = 341)		
Yes	188	55.1
No	153	44.9
Ignored (n = 938)	597	63.6
Refused chemoprophylaxis (n = 231)		
Yes	16	19.2
No	215	80.8
Ignored (n = 938)	707	75.4
Human immunoglobulin against hepatitis B (n = 211)		
Yes	23	10.9
No	188	89.1
Ignored (n = 938)	727	77.5
Vaccine against hepatitis B (n=251)		
Yes	69	27.5
No	182	72.5
Ignored (n = 938)	687	73.2

The clinical progression of victims of occupational accidents involving biological material (n = 938) between 2007 and 2016 in Amapá had 91.9% of the data reported as unknown or blank. However, among the 76 followed-up cases, the most frequent outcome was discharge with no serological conversion (47.4%), whereas 15.8% of the patients were discharged with serological conversion (Table 5).

**Table 5:** Clinical progression of victims of occupational accidents involving biological material (n = 938) between 2007 and 2016 in the state of Amapá, Brazil

Clinical progression*	n	%
Non-adherence	3	3.9
Discharge with serological conversion	12	15.8
Discharge with negative source patient	24	31.6
Discharge without serological conversion	36	47.4
Death due to other causes	1	1.3
Unknown/blank (n = 938)	862	91.9
Total	938	100.0

* n = 76 followed-up cases.

## DISCUSSION

The hospital environment stood out as a notifying agent, especially in the capital Macapá, which may be associated with a greater density of workers and the execution of invasive procedures, highlighting the need for continued education on accident prevention, biosecurity, and the use of PPE in these environments. PPE use provides the worker with a safe source of protection against the exposure to biological material.^1^ A study by Corrêa et al.^12^ indicated gloves as the most used piece of PPE when compared to other items. These authors associated a low use of PPE with a resistance by professionals and a low availability of equipment in work environments. According to Vieira et al.^13^, the inadequate disposal in inappropriate and full containers contributes to the occurrence of accidents involving biological material.

Regarding the importance of knowing the serological status of the source patient, Carneiro and Cordeiro et al.^11^ stated that serological positivity for HIV in the source patient should lead to the immediate recommendation of antiretroviral drugs, just as negativity for this virus leads to the contraindication of chemoprophylaxis. Recommendations of vaccination and immunoglobulin against hepatitis B are performed based on the knowledge of the serological status of the source patient. In case the victim of the occupational accident presents a positive vaccine response,^11^ the authors highlight the need for recognizing the serological status for hepatitis C, since this disease still does not have a post-exposure drug recommendation; prevention thus constitutes the best conduct.

Notification is a fundamental step in the follow-up of an individual who has suffered an occupational accident. However, in this study, many items were marked as unknown/blank, which did not allow us to understand the reality of health care institutions. In the study conducted by Santos Junior et al.,^14^ both companies and professionals resisted to the notification process, which could be related to a disbelief or lack of knowledge on post-incident follow-up. The notifications of occupational accidents involving biological material in the state of Amapá have grown during the evaluated period; however, we believe that registers are still underreported. The main type of exposure was percutaneous; the most reported organic material was blood; and the main causative agent was the hollow-bore needle. The main circumstances at the moment of the accident were the incorrect disposal of sharps and intravenous drug administration. Out of the patients who received recommendations for HIV chemoprophylaxis, only 2% (16) accepted the treatment. Recommendations for human immunoglobulin against hepatitis B and vaccination against hepatitis B were reported, respectively, in 23 (2.5%) and 69 (7%) cases. The discrepancy between patients with a chemoprophylaxis recommendation and those who accepted treatment is probably due to the partial register of the notification record. Most patients had unknown or unrecorded data on post-incident follow-up. When considering followed-up cases, the most frequent outcome was discharge with no serological conversion; in case of discharge with serological conversion, we could not identify the causative infectious agent. We identified many cases recorded as unknown/blank regarding the vaccination profile against hepatitis B and anti-HIV, HBsAg, anti-HBs, and anti-HCV serological profiles.

This study demonstrated that most accidents occurred during handling of sharps, indicating inadequate or careless handling as one of the main risk factors for accidents involving biological material. Flaws in the recording process were also observed considering the need for training professionals that execute procedures involving sharps, the use of safety devices by these workers, and the organization of the correct disposal of these materials; these data are important for patient follow-up. Moreover, this study identified the need to strengthen the notification network in order for us to have a more accurate view of this problem and its real impact on health workers. Therefore, this study indicates the need for a periodic assessment of notifications so they can be used as a basis for improving the epidemiological surveillance system and prevention policies.
